# Supplementary material for: Unveiling the Bioactive Potential of the Invasive Jellyfish Phyllorhiza punctata Through Integrative Transcriptomic and Proteomic Analyses
Source: Biomolecules. 2025 Aug 4;15(8):1121. doi: 10.3390/biom15081121 (PMC12383608; doi:10.3390/biom15081121)
Supplement: Supplementary file 1 [file biomolecules-15-01121-s001.zip › Suplementary Material/FileS3/PEPFOLD4/XP_065072400_12624/clusters.html]

 PEP-FOLD clusters


Download archive of all models  here   

|  |  |  |
| --- | --- | --- |
| # | file | sOPEP |
| # cluster 1 - Size: 17 |  |  |
|  | XP\_065072400\_12624-model1 | -25.3634 |
|  | XP\_065072400\_12624-model1.1 | -25.3005 |
|  | XP\_065072400\_12624-model1.2 | -25.2309 |
|  | XP\_065072400\_12624-model1.3 | -24.9265 |
|  | XP\_065072400\_12624-model1.4 | -24.8765 |
|  | XP\_065072400\_12624-model1.5 | -24.856 |
|  | XP\_065072400\_12624-model1.6 | -24.7914 |
|  | XP\_065072400\_12624-model1.7 | -24.7846 |
|  | XP\_065072400\_12624-model1.8 | -24.6589 |
|  | XP\_065072400\_12624-model1.9 | -24.6589 |
|  | XP\_065072400\_12624-model1.10 | -24.4225 |
|  | XP\_065072400\_12624-model1.11 | -24.3739 |
|  | XP\_065072400\_12624-model1.12 | -24.2188 |
|  | XP\_065072400\_12624-model1.13 | -24.0085 |
|  | XP\_065072400\_12624-model1.14 | -23.8998 |
|  | XP\_065072400\_12624-model1.15 | -23.7357 |
|  | XP\_065072400\_12624-model1.16 | -23.0227 |
| # cluster 2 - Size: 8 |  |  |
|  | XP\_065072400\_12624-model2 | -25.0987 |
|  | XP\_065072400\_12624-model2.1 | -24.5006 |
|  | XP\_065072400\_12624-model2.2 | -24.4848 |
|  | XP\_065072400\_12624-model2.3 | -24.2539 |
|  | XP\_065072400\_12624-model2.4 | -24.1296 |
|  | XP\_065072400\_12624-model2.5 | -23.8368 |
|  | XP\_065072400\_12624-model2.6 | -22.9975 |
|  | XP\_065072400\_12624-model2.7 | -22.7115 |
| # cluster 3 - Size: 6 |  |  |
|  | XP\_065072400\_12624-model3 | -25.0345 |
|  | XP\_065072400\_12624-model3.1 | -25.0345 |
|  | XP\_065072400\_12624-model3.2 | -24.2357 |
|  | XP\_065072400\_12624-model3.3 | -23.9314 |
|  | XP\_065072400\_12624-model3.4 | -23.5678 |
|  | XP\_065072400\_12624-model3.5 | -22.8755 |
| # cluster 4 - Size: 4 |  |  |
|  | XP\_065072400\_12624-model4 | -25.0159 |
|  | XP\_065072400\_12624-model4.1 | -24.7639 |
|  | XP\_065072400\_12624-model4.2 | -24.4209 |
|  | XP\_065072400\_12624-model4.3 | -22.7304 |
| # cluster 5 - Size: 10 |  |  |
|  | XP\_065072400\_12624-model5 | -23.581 |
|  | XP\_065072400\_12624-model5.1 | -21.5792 |
|  | XP\_065072400\_12624-model5.2 | -21.0962 |
|  | XP\_065072400\_12624-model5.3 | -20.951 |
|  | XP\_065072400\_12624-model5.4 | -20.7029 |
|  | XP\_065072400\_12624-model5.5 | -20.4648 |
|  | XP\_065072400\_12624-model5.6 | -20.4348 |
|  | XP\_065072400\_12624-model5.7 | -20.3706 |
|  | XP\_065072400\_12624-model5.8 | -20.3396 |
|  | XP\_065072400\_12624-model5.9 | -19.891 |
| # cluster 6 - Size: 4 |  |  |
|  | XP\_065072400\_12624-model6 | -22.8088 |
|  | XP\_065072400\_12624-model6.1 | -21.0212 |
|  | XP\_065072400\_12624-model6.2 | -19.2827 |
|  | XP\_065072400\_12624-model6.3 | -19.0021 |
| # cluster 7 - Size: 2 |  |  |
|  | XP\_065072400\_12624-model7 | -22.6876 |
|  | XP\_065072400\_12624-model7.1 | -20.7708 |
| # cluster 8 - Size: 1 |  |  |
|  | XP\_065072400\_12624-model8 | -22.3716 |
| # cluster 9 - Size: 2 |  |  |
|  | XP\_065072400\_12624-model9 | -21.8338 |
|  | XP\_065072400\_12624-model9.1 | -20.5836 |
| # cluster 10 - Size: 4 |  |  |
|  | XP\_065072400\_12624-model10 | -21.171 |
|  | XP\_065072400\_12624-model10.1 | -20.8805 |
|  | XP\_065072400\_12624-model10.2 | -20.5581 |
|  | XP\_065072400\_12624-model10.3 | -19.5859 |
| # cluster 11 - Size: 1 |  |  |
|  | XP\_065072400\_12624-model11 | -20.7206 |
| # cluster 12 - Size: 1 |  |  |
|  | XP\_065072400\_12624-model12 | -20.2109 |
| # cluster 13 - Size: 1 |  |  |
|  | XP\_065072400\_12624-model13 | -20.2038 |
| # cluster 14 - Size: 6 |  |  |
|  | XP\_065072400\_12624-model14 | -19.7867 |
|  | XP\_065072400\_12624-model14.1 | -19.1196 |
|  | XP\_065072400\_12624-model14.2 | -19.1109 |
|  | XP\_065072400\_12624-model14.3 | -18.66 |
|  | XP\_065072400\_12624-model14.4 | -18.3615 |
|  | XP\_065072400\_12624-model14.5 | -18.2391 |
| # cluster 15 - Size: 6 |  |  |
|  | XP\_065072400\_12624-model15 | -19.5197 |
|  | XP\_065072400\_12624-model15.1 | -19.2074 |
|  | XP\_065072400\_12624-model15.2 | -18.9725 |
|  | XP\_065072400\_12624-model15.3 | -18.7799 |
|  | XP\_065072400\_12624-model15.4 | -18.4945 |
|  | XP\_065072400\_12624-model15.5 | -18.2994 |
| # cluster 16 - Size: 3 |  |  |
|  | XP\_065072400\_12624-model16 | -19.4299 |
|  | XP\_065072400\_12624-model16.1 | -18.8311 |
|  | XP\_065072400\_12624-model16.2 | -17.8302 |
| # cluster 17 - Size: 1 |  |  |
|  | XP\_065072400\_12624-model17 | -18.7483 |
| # cluster 18 - Size: 3 |  |  |
|  | XP\_065072400\_12624-model18 | -18.443 |
|  | XP\_065072400\_12624-model18.1 | -17.9825 |
|  | XP\_065072400\_12624-model18.2 | -17.8568 |
| # cluster 19 - Size: 1 |  |  |
|  | XP\_065072400\_12624-model19 | -18.4078 |
| # cluster 20 - Size: 2 |  |  |
|  | XP\_065072400\_12624-model20 | -18.2888 |
|  | XP\_065072400\_12624-model20.1 | -18.2838 |
| # cluster 21 - Size: 2 |  |  |
|  | XP\_065072400\_12624-model21 | -18.2346 |
|  | XP\_065072400\_12624-model21.1 | -18.1981 |
| # cluster 22 - Size: 13 |  |  |
|  | XP\_065072400\_12624-model22 | -18.22 |
|  | XP\_065072400\_12624-model22.1 | -18.2127 |
|  | XP\_065072400\_12624-model22.2 | -18.2097 |
|  | XP\_065072400\_12624-model22.3 | -18.2075 |
|  | XP\_065072400\_12624-model22.4 | -18.0876 |
|  | XP\_065072400\_12624-model22.5 | -18.0678 |
|  | XP\_065072400\_12624-model22.6 | -18.024 |
|  | XP\_065072400\_12624-model22.7 | -18.0158 |
|  | XP\_065072400\_12624-model22.8 | -17.9807 |
|  | XP\_065072400\_12624-model22.9 | -17.8875 |
|  | XP\_065072400\_12624-model22.10 | -17.8573 |
|  | XP\_065072400\_12624-model22.11 | -17.7842 |
|  | XP\_065072400\_12624-model22.12 | -17.7582 |
| # cluster 23 - Size: 2 |  |  |
|  | XP\_065072400\_12624-model23 | -18.0648 |
|  | XP\_065072400\_12624-model23.1 | -18.04 |
|  |  |  |
